# Store-operated calcium entry facilitates LPS-induced superoxide anion-dependent macrophage extracellular traps

**DOI:** 10.1098/rsob.250024

**Published:** 2025-07-09

**Authors:** Thang Ngoc Nguyen, Tzu-Chien Lin, Waratchaya Chimphlee, Kon Xuen Siew, Naphatsawan Vongmanee, Shih-Chuan Hsiao, Sarinporn Visitsattapongse, Wen-Tai Chiu

**Affiliations:** ^1^ Department of Biomedical Engineering, National Cheng Kung University, Tainan City, Taiwan; ^2^ Department of Biomedical Engineering, King Mongkut’s Institute of Technology Ladkrabang, Bangkok, Thailand; ^3^ Department of Hematology and Oncology, Saint Martin de Porres Hospital, Chiayi, Taiwan; ^4^ International Center for Wound Repair and Regeneration, National Cheng Kung University, Tainan City, Taiwan; ^5^ Medical Device Innovation Center, National Cheng Kung University, Tainan City, Taiwan

**Keywords:** NETosis, METosis, calcium ions (Ca^2+^), NADPH oxidases, store-operated Ca^2+^ entry, superoxide anion

## Abstract

Macrophage extracellular traps (METs) represent a recently discovered complex defence mechanism that is distinct from phagocytosis and involves the release of DNA and antibacterial proteins. They play an important role in pathogen removal, and calcium ions (Ca^2+^) have also been reported to be involved. In the present study, we identified METotic cells using digitonin as an alternative to Triton X-100, coupled with immunofluorescence staining using lamin antibodies. The limited permeability of digitonin ensures exclusive intranuclear antibody labelling of MET cells, therefore providing a straightforward and intuitive differentiation method. We found that under lipopolysaccharide stimulation, macrophages undergo store-operated Ca^2+^ entry (SOCE) to facilitate Ca^2+^ influx. Elevation of cytoplasmic Ca^2+^ levels by SOCE promotes the generation of superoxide anions by NADPH oxidase (NOX), ultimately leading to METosis. In summary, our study strengthens the role of Ca^2+^ in NOX-dependent METosis, which differs from previous studies focusing on Ca^2+^ in the NOX-independent pathway. Our research reveals that Ca^2+^-mediated regulation of NOX plays a crucial role in METosis, especially in SOCE, and provides novel ideas for future research.

**Figure:**
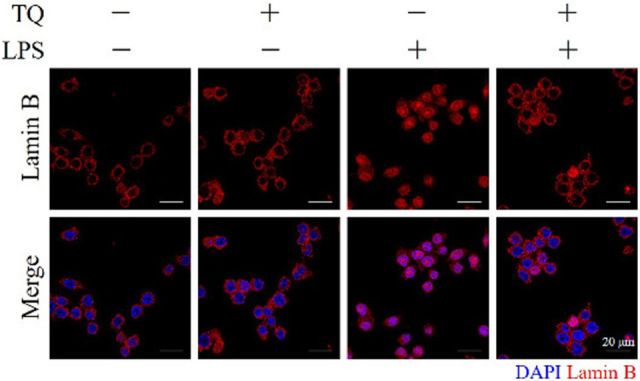

## 1. Introduction

Neutrophils and macrophages combat infections through phagocytosis, degranulation and extracellular traps [1], a concept officially named neutrophil extracellular traps (NETs). Furthermore, certain common irritants, such as phorbol myristate acetate (PMA), lipopolysaccharide (LPS) or interleukin-8 [2,3], were listed. Stimulated neutrophils undergo nuclear deformation, chromatin homogenization and subsequent rupture of nuclear and granular membranes, releasing NETs, which are complex structures composed of chromatin, histones and antimicrobial proteins. They form an external network, deploy antimicrobial agents to neutralize trapped microbes and initiate a local immune response to recruit additional immune cells. Extracellular traps occur not only in neutrophils but also in other immune cells, such as mast cells [4] and macrophages [5]. Similar to neutrophils, monocytes and macrophages have been shown to release extracellular traps in a process called METosis. METs mainly work in immuno-response related to the cell death of pathogenic microorganism, such as in necroptosis, tumour or AKI. Under normal physiological conditions, macrophages form METs to capture and kill pathogens when they receive external stimuli, thus exerting innate immune function [6]. Necroptosis induces METs to promote pancreatic ductal adenocarcinoma liver metastasis through degradation of the extracellular matrix [7]. In addition, METs formation is also a key mediator of rhabdomyolysis-induced acute kidney injury [8].

NADPH is used in the enzymatic reactions in reducing oxidized glutathione (GSSG) to its reduced form (GSH) [9]. GSH is ubiquitous in mammalian cells and an essential non-enzymatic antioxidant that eliminates free radicals [10]. NADPH oxidase (NOX) is a membrane-bound enzyme complex that is important for the detoxification of the pathogen after phagocytosis of immune cells; NADPH is oxidized by NOX to produce superoxide anions [11]. NADPH is continuously oxidized and reduced to form a series of reactive oxygen species (ROS) [12]. Excessive ROS production can cause tissue damage and inflammation. Thymoquinone (TQ), is a potential scavenger of superoxide anions [13], and could be used against oxidative stress [14]. Numerous studies have consistently demonstrated that the activation of neutrophils by the potent inducer PMA triggers the protein kinase C pathway, resulting in changes in intracellular Ca^2+^ flow and the activation of NOX, ultimately resulting in the generation of ROS [15,16]. The inhibition of NOX activity reduces NETosis and mitigates thrombotic mass formation in murine models of heparin-induced thrombocytopenia [17]. Current investigations predominantly emulate the established mechanisms of NETs, notably involving NOX [8]. The NET formation pathways can be roughly divided into dependent and independent NADPH/NOX [18]. However, most studies rely on NOX-derived ROS to treat NETosis [19].

Calcium ions (Ca^2+^) assume the critical role of a signalling molecule that intricately regulates various immune cell activities. During the inflammatory response, mitochondria respond to Ca^2+^-dependent stress signals and produce ROS in bone marrow-derived macrophages [20]. Moreover, Ca^2+^ via the activation of the Ca^2+^-sensing receptor in immune cells accumulates in plaques and secretes pro-inflammatory cytokines, amplifying the local inflammatory response [21,22]. Ca^2+^ also plays a crucial role in the formation of extracellular traps [23]. Store-operated Ca^2+^ entry (SOCE) is the main mechanism regulating intracellular Ca^2+^ stability [24]. This regulates the influx of Ca^2+^ from the extracellular space into the cytoplasm in response to the depletion of Ca^2+^ stores in the endoplasmic reticulum (ER) [25]. After the extramembrane receptor receives an external signal, activated phospholipase C hydrolyses phosphatidylinositol 4,5-bisphosphate into diacylglycerol and inositol 1,4,5-trisphosphate. This in turn binds to the inositol 1,4,5-trisphosphate receptor on the ER and promotes the outflow of Ca^2+^ from the ER, thereby increasing the Ca^2+^ concentration in the cytoplasm [26]. Reduced ER Ca^2+^ leads to the accumulation of STIM1, an ER Ca^2+^ sensor, on the ER membrane and binding with ORAI1 or TRPC1 Ca^2+^ channels on the cell membrane, allowing Ca^2+^ influx [27]. This pathway is widespread in eukaryotic cells and influences various cellular functions [28]. In the present study, we aimed to elucidate the regulatory role of Ca^2+^ in MET genesis, decipher the potential involvement of NOX/ROS and delineate upstream and downstream relationships within this intricate process.

## 2. Material and methods

### 2.1. Cell culture

RAW264.7 cells (mouse macrophages) and U937 cells (human monocytes) were maintained in high-glucose Dulbecco’s Modified Eagle Medium (Caisson, DMP27) and RPMI-1640 Medium (Thermo Fisher Scientific, 11875093), respectively. The cells were also supplemented with 10% fetal bovine serum (FBS; Gibco, A5256701) and penicillin–streptomycin (P/S; Caisson Laboratories, PSL01) under 5% CO_2_ at 37°C.

### 2.2. Chemical treatment

All experiments were performed after seeding and incubation for 24 h. For pretreatment, the Ca^2+^ chelator 20 μM BAPTA-AM (Sigma, A1076), the mechanical Ca^2+^ channel inhibitor 50 μM GdCl_3_ (Sigma, G7532), the SOCE inhibitors 2 μM SKF96365 (Cayman, 10 009 312) and 10 μM YM58483 (Cayman, 13246) and the superoxide anion scavenger 5 μM thymoquinone (TQ; Sigma, AL-274666) were added for 30 min. Thereafter, the cells were treated with 500 nM phorbol 12-myristate 13-acetate (PMA; Sigma, P8139) or 1 μg ml^−1^ LPS (Enzo Life Sciences, ALX-581-013) for 3 h.

### 2.3. Immunofluorescence staining

RAW264.7 cells were seeded at a concentration of 1.5 × 10^5^ in a 3 cm glass-bottomed dish (Alpha Plus, 16235-1SGP15). After treatment, 4% paraformaldehyde (PFA; Boster, AR1068) was added to the fixed cells for 10 min and permeated with 20 μM digitonin (Sigma, D141) for 10 min, and the CAS-block buffer (Invitrogen, 008120) was also used for 1 h at 25°C. The cells were then incubated overnight at 4°C with the primary antibody, lamin B (Abclonal, A16909). After washing with PBS for 1 h, Alexa Fluor 488- or Alexa Fluor 594-conjugated secondary antibodies (Invitrogen) were diluted in PBS and 4′,6-diamidino-2-phenylindole (DAPI) (GeneTex, GTX16206). All images were captured using an Olympus confocal microscope (FV3000).

### 2.4. Nucleus and cytosol fractionation

RAW264.7 cells were seeded at a concentration of 2 × 10^6^ in a 10 cm culture dish. All cells were collected, the medium was centrifuged to remove the supernatant, and 1 ml of cold PBS was added to resuspend the cells, which were then transferred to an Eppendorf tube. Subsequent preparations were performed on ice. The refrigerated centrifuge was used to centrifuge the cell solution (1200 rcf, 3 min, 4°C), the supernatant was removed, 200 μl hypotonic buffer (20 mM Tris-HCl, 10 mM NaCl, 3 mM MgCl_2_) was gently added to resuspend cells, and placed on ice for 15 min. Twenty microlitres of 10% NP-40 was added, vortexed for 10 s, and centrifuged to pellet nuclei (3000 r.p.m., 10 min, 4°C). The supernatant (cytosolic fraction) was collected, and the pellet was recentrifuged twice after adding hypotonic buffer to remove the residual cytoplasm. The pellets were incubated on ice for 30 min and vortexed every 10 min after adding 60 μl radioimmunoprecipitation lysis buffer. After centrifugation (13 000 rcf, 10 min, 4°C), the supernatant (nuclear fraction) was collected and the collected fractions were frozen at −80°C.

### 2.5. Western blotting

Cell lysates were collected in a radioimmunoprecipitation assay buffer on ice. The protein samples were mixed with the buffer and denatured by heating at 95°C for 10 min. Thereafter, the samples were electrophoresed on 10% SDS-PAGE and were transferred onto nitrocellulose membranes, blocked with 5% skimmed milk diluted in washing buffer (0.4 M Tris, 1 M NaCl, 0.05% Twenty-20) for 1 h. The membranes were incubated at 4°C overnight with the following primary antibodies: lamin B (Abclonal, A16909), NOX1 (abcam, ab131088), NOX2 (abcam, ab310337) and GAPDH (abcam, ab8245). After extensive washing with washing buffer for 1 h, secondary antibodies were added to horseradish peroxidase-conjugated IgG at room temperature for 1 h. Finally, the membranes were enhanced using an ECL Substrate Kit (Abcam, ab133406) and images were captured using the Amersham Imager 600 system.

### 2.6. Single-cell intracellular Ca^2+^ measurement

The cells were incubated with 2 μM Fura-2AM (Invitrogen, F1221) for 30 min to record the real-time changes in intracellular Ca^2+^ after PMA or LPS stimulation. Next, 0 mM Ca^2+^ buffer or 2 mM Ca^2+^ buffer was added to measure Ca^2+^ influx into the cytosol. Fura-2 is a fluorescent Ca^2+^ probe with two excitation wavelengths. If bonded to Ca^2+^, it generates minimal emission at 510 nm under excitation at 380 nm, and vice versa; it generates maximal emission at 510 nm under excitation at 340 nm. The ratio of the fluorescence intensities at 510 nm excited at 340 and 380 nm (F340/F380) indicated the Ca^2+^ concentration in the cytosol, as detected by single-cell fluorimetry (TILL Photonics).

### 2.7. Real-time extracellular trap formation in macrophage

Cells were seeded at a concentration of 1.5× 10^5^ in a 3 cm glass-bottomed dish for 24 h, incubated with DAPI for 30 min, following which 5  μM YO-PRO-1 with or without 1 μg ml^−1^ LPS was added. The dishes were placed in a mini-incubator under 5% CO_2_ at 37°C to maintain their viability and photographed every 5 min using an inverted fluorescence microscope (Olympus IX71).

### 2.8. Detection of reactive oxygen species

The Cellular ROS Assay Kit, 10 μM dichlorodihydrofluorescein diacetate (DCFDA; abcam, ab113851), or the superoxide anion detector, 5 μM dihydroethidium (DHE; Sigma-Aldrich, D7008), was used with DAPI for 30 min after treatment. All fluorescent probes were imaged using an inverted fluorescence microscope (Olympus IX71).

### 2.9. Statistics

All experiments were repeated at least thrice, and the results were reproducible. One-way ANOVA was used to determine statistically significant differences between independent groups. Post hoc analysis was conducted using Tukey’s test. SPSS Statistics 17.0 and Origin software were used to perform statistical analyses (*p*-values: # or *<0.05, ## or **<0.01, ### or ***<0.001).

## 3. Results

### 3.1. Identification of PMA- or LPS-induced METs in macrophages

**Figure 1.**
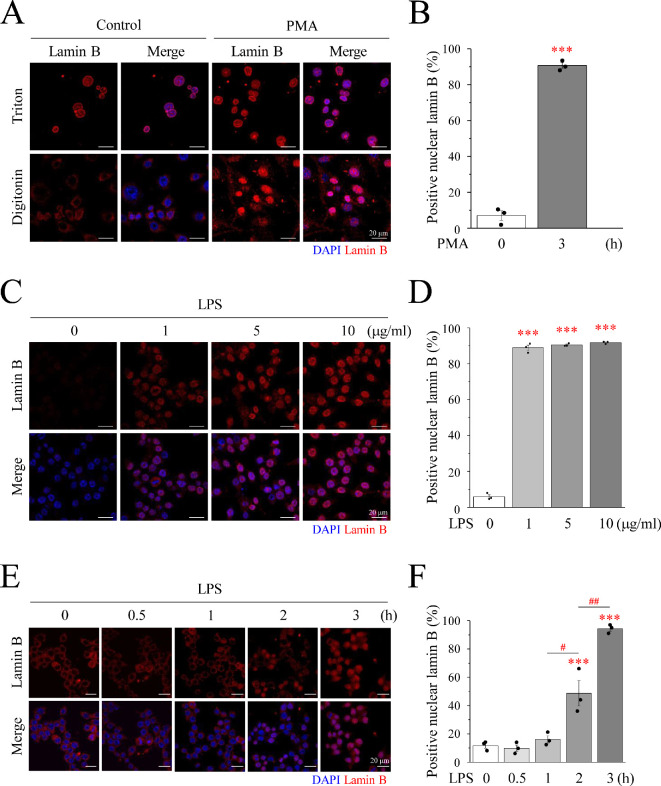
LPS induces METosis in a time-dependent manner. RAW264.7 cells were treated with (A,B) PMA or (C–F) LPS for 3 h. Cells were fixed with 4% paraformaldehyde in PBS for 10 min and permeabilized with Triton X-100 or digitonin for 10 min before blocking. (A,C,E) Immunofluorescence images of DAPI (blue) and lamin B (red) staining. (C) RAW264.7 cells were treated with 1, 5 or 10 μg ml^−1^ LPS for 3 h, or (E) 1 μg ml^−1^ LPS for 0.5, 1, 2, 3 h. Scale bar: 20 μm. (B,D,F) Percentage of cells positive for nuclear lamin B staining. PMA: phorbol myristate acetate; LPS: lipopolysaccharide; DAPI: DAPI: 4′,6-diamidino-2-phenylindole.

The rupture of the nuclear membrane and release of DNA molecules are characteristics of METosis. The response of METosis to PMA, a well-known MET inducer, was examined in mouse macrophage RAW264.7 cells. Two different immunofluorescence staining procedures were tested to determine the most suitable method for MET observation: Triton X-100 or digitonin permeabilization after fixing the cells with 4% paraformaldehyde. Triton X-100, a non-ionic surfactant, can dissolve lipids in cell, nuclear and organellar membranes. Because digitonin, a steroidal saponin, acts only on the cell membrane and not on the nuclear membrane, it is best able to identify the rupture of the MET nuclear membrane and split DNA into the cytoplasm or even outside the cell (figure 1A). We found that digitonin, but not Triton X-100, is the easiest option for determining whether PMA causes MET (figure 1B). After permeabilization with digitonin, if the lamin B antibody stains positively in the nucleus, the cells undergo METosis. However, PMA is an artificial product and is not a substance present in the body. LPS, a component of the outer membrane of Gram-negative bacteria, is also used as an inducer that exists in the body and causes METosis. The most appropriate concentration and duration of LPS action were determined in a dose- and time-dependent manner. The lowest concentration of 1 μg ml^−1^ LPS already had a significant effect, and increasing the concentration did not significantly increase its effect (figure 1C,D). In time-dependent experiments, LPS significantly induced METosis in approximately 50% of cells in just 2 h. When the action time was increased to 3 h, METosis increased to approximately 90%. Therefore, in subsequent experiments, 1 μg ml^−1^ LPS treatment for 3 h was selected (figure 1E,F).

**Figure 2.**
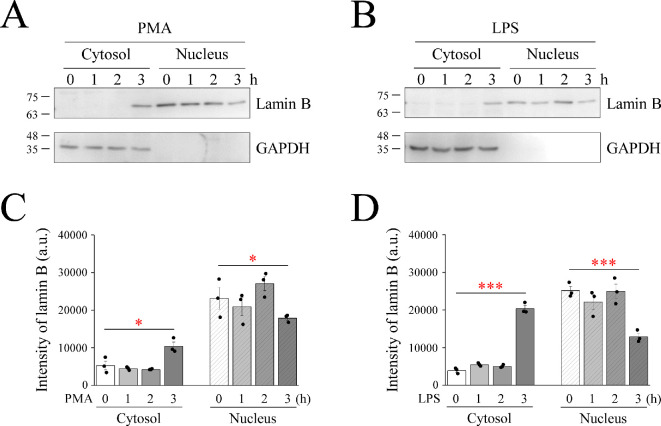
LPS treatment leads to lamin B release to cytosol. RAW264.7 cells were treated with PMA or LPS for 3 h. Representative immunoblotting with lamin B and GAPDH from the cytosol/nuclear fraction of (A) PMA or (B) LPS. GAPDH served as an internal control. Intensity of lamin B after (C) PMA or (B) LPS treatment. GAPDH: glyceraldehyde-3-phosphate dehydrogenase. Other abbreviations are the same as given in the legend to figure 1.

Real-time imaging was performed to evaluate the progression of LPS-induced METosis. YO-PRO-1 is a green fluorescent cell-impermeant nucleic acid dye, whereas Hoechst 33 342 is a blue fluorescent cell-permeant nucleic acid dye. When the cell membrane leaks or ruptures, YO-PRO-1 enters the cell nucleus and stains the DNA. The LPS effect group began to appear green as early as 30 min and gradually increased in colour until the end of the shooting period (electronic supplementary material, figure S1). In addition, nuclear and cytoplasmic separation combined with Western blotting were used to further confirm the formation of METs. After 3 h of exposure to PMA or LPS, lamin B, a nuclear substance that should normally not be present, was detected in the cytoplasm (figure 2). In the present study, we successfully established an experimental model for inducing and examining MET.

### 3.2. Store-operated Ca^2+^ entry was found essential for LPS-induced METosis

**Figure 3.**
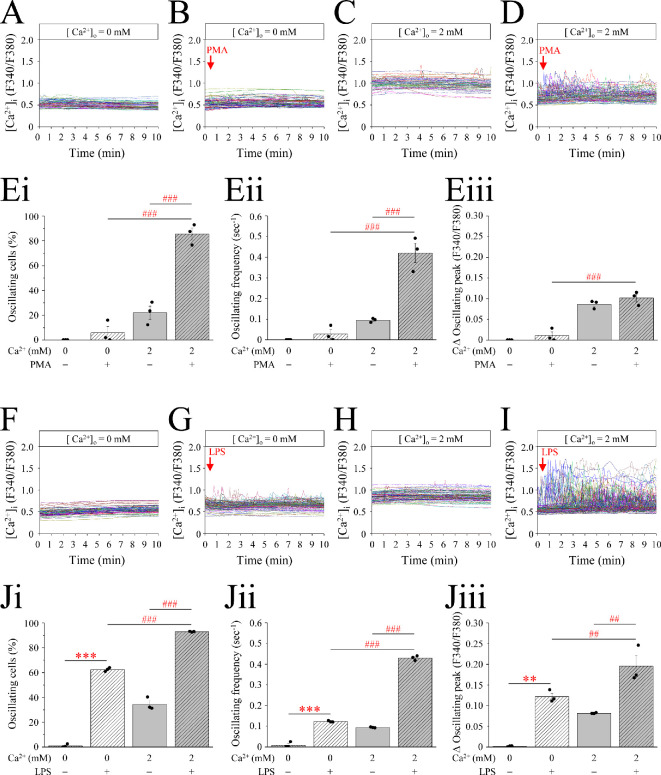
PMA and LPS trigger extracellular Ca^2+^ influx in macrophages. RAW264.7 cells were pre-treated with the fluorescent Ca^2+^ probe Fura-2AM for 30 min. Ca^2+^ signalling was measured at alternating excitation wavelengths of 340 and 380 nm for 10 min. (A,F) Intracellular Ca^2+^ concentrations in Ca^2+^-free buffer (0 mM). (B,G) Intracellular Ca^2+^ concentration in Ca^2+^-free buffer (0 mM), and PMA or LPS was added in the first minute. (C,H) Intracellular Ca^2+^ concentration in 2 mM Ca^2+^ buffer. (D,I) Intracellular Ca^2+^ concentration in 2 mM Ca^2+^ buffer, and PMA or LPS was added during the 30 s. Bar charts represent the percentage of oscillating cells (E(i) and J(i)), frequency of the oscillating cells (E(ii) and J(ii)) and amplitude of the Ca^2+^ level (E(iii) and J(iii)). Abbreviations are the same as given in the legend to figure 1.

PMA induced an increase in intracellular Ca^2+^ concentration. Using a single-cell fluorometer in conjunction with Fura-2AM, a ratiometric fluorescent Ca^2+^ probe was designed for cytosolic Ca^2+^ measurement. As depicted in figure 3, unstimulated cells exhibited minimal signal fluctuations in both Ca^2+^-free (figure 3A) and 2 mM Ca^2+^ media (figure 3C), approximating baseline levels. However, upon the addition of PMA to cells in 2 mM Ca^2+^ medium in the first minute, substantial and prolonged fluctuations in intracellular Ca^2+^ signals occurred (figure 3D). The percentage of oscillating cells (figure 3E(i)), frequency (figure 3E(ii)) and amplitude (figure 3E(iii)) of these oscillations markedly surpassed those in the untreated group. In contrast, in the Ca^2+^-free medium group characterized by the absence of extracellular Ca^2+^, the observed change was inconspicuous and nearly negligible (figure 3B). These data showed that the Ca^2+^ oscillations caused by PMA mainly originate from extracellular Ca^2+^ influx. Moreover, LPS, which induces METosis in the presence of infectious bacteria in the body, was also used to verify its effect on Ca^2+^ signalling. In the absence of LPS in both Ca^2+^-free (figure 3F) and 2 mM Ca^2+^ media (figure 3H), the lack of Ca^2+^ oscillation within the cells was consistent with PMA treatment. Nevertheless, upon the introduction of LPS into a 2 mM Ca^2+^ medium environment, a conspicuous oscillation emerged (figure 3I) in terms of the percentage of oscillating cells (figure 3J(i)), frequency (figure 3J(ii)) and amplitude (figure 3J(iii)). The results of this experiment revealed more pronounced alterations compared with those obtained with PMA treatment. Notably, in contrast to the almost negligible changes observed after PMA stimulation, the group exposed to Ca^2+^-free medium displayed significant Ca^2+^ oscillations following LPS stimulation (figure 3G). However, the signal was still inferior to that in the extracellular environment with physiological 2 mM Ca^2+^ (figure 3J).

**Figure 4.**
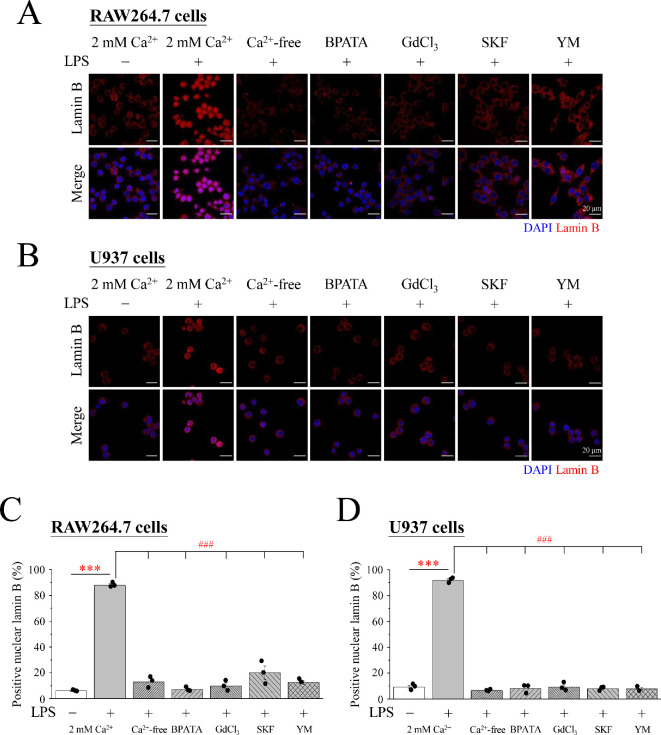
SOCE is essential for LPS-induced METosis in RAW264.7 and U937 cells. RAW264.7 and U937 cells were treated with 1 μg ml^−1^ LPS for 3 h in Ca^2+^-free (0 mM) or 2 mM Ca^2+^ medium. In addition, pre-treatment with BAPTA-AM, GdCl_3_, SKF96365 or YM58483 was carried out for 30 min and then with LPS for 3 h. (A,B) Immunofluorescence images of DAPI (blue) and lamin B (red). Scale bar: 20 μm. (C,D) The percentage of cells that stained positive for nuclear lamin B. SOCE: store-operated Ca^2+^ entry; LPS: lipopolysaccharide; DAPI: 4′,6-diamidino-2-phenylindole.

The role of Ca^2+^ in LPS-induced METs formation was examined using several chemicals, including the Ca^2+^ chelator BAPTA-AM, the non-specific and efficacious Ca^2+^ channel blocker GdCl_3_ and the SOCE inhibitors SKF96365 and YM58483 (figure 4). Notably, LPS significantly triggered METs in 2 mM Ca^2+^ medium in both RAW264.7 and U937 cells. After treating the cells with LPS in Ca^2+^-free medium, nuclear lamin B was not stained, indicating that it significantly reduced the production of METs. The cells were pre-treated with BAPTA-AM, GdCl_3_, SKF96365 or YM58483 in 2 mM Ca^2+^ medium before adding LPS for stimulation, which reduced the staining of lamin B in the nucleus induced by LPS (figure 4). Immunofluorescence staining showed that in the absence of Ca^2+^, Ca^2+^-free medium and BAPTA significantly inhibited METosis. Inhibiting the influx of Ca^2+^, whether fully inhibited by GdCl_3_ or the SOCE inhibitors YM58483 and SKF96365, effectively reduced MET production.

### 3.3. SOCE affected LPS-induced superoxide anion generation of METosis by activating NADPH oxidases

**Figure 5.**
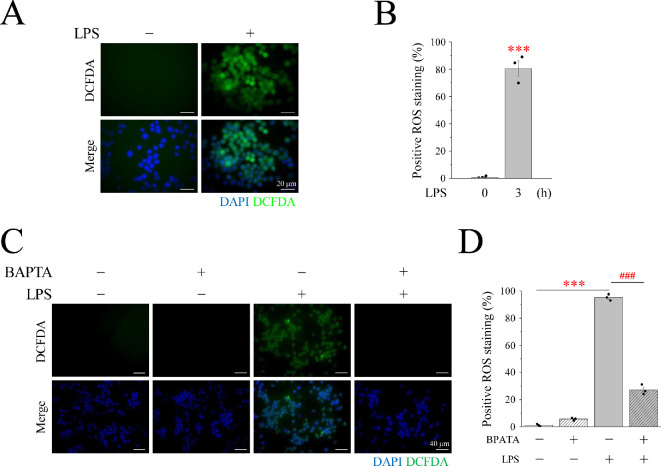
Intracellular Ca^2+^ is essential for cells to produce ROS by LPS treatment. (A) RAW264.7 cells were treated with 1 μg ml^−1^ LPS for 3 h. Fluorescence imaging of DAPI (blue) and DCFDA (green). Scale bar: 20 μm. (C) RAW264.7 cells were pre-treated BAPTA-AM for 30 min, then with 1 μg ml^−1^ LPS for 3 h. Fluorescence imaging of DAPI (blue) and DCFDA (green). Scale bar: 40 μm. (B,D) The percentage of cells with positive ROS staining. ROS: reactive oxygen species; LPS: lipopolysaccharide; DAPI: 4′,6-diamidino−2-phenylindole; DCFDA: dichlorodihydrofluorescein diacetate.

**Figure 6.**
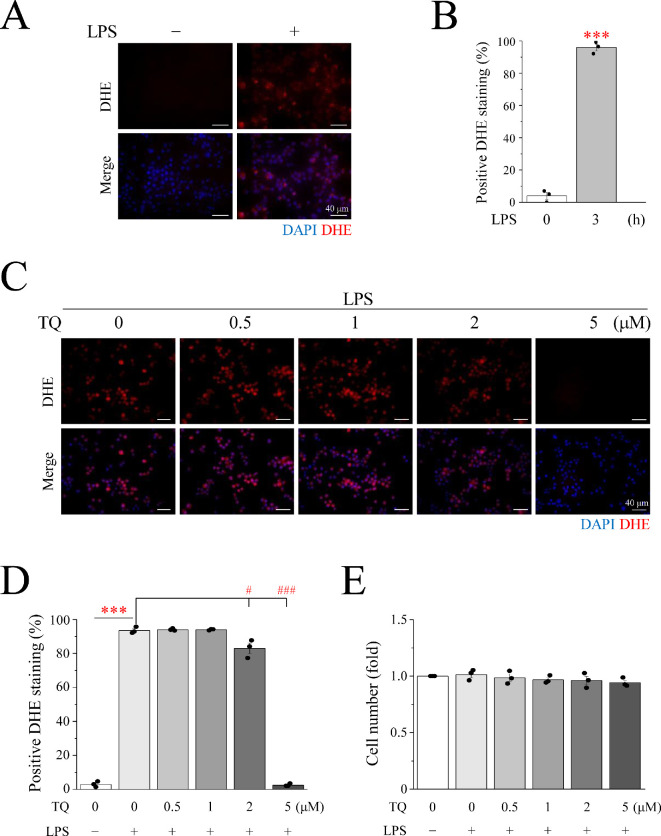
LPS treatment leads to superoxide anion production. (A) RAW264.7 cells were treated with 1 μg ml^−1^ LPS for 3 h. Fluorescence imaging of DAPI (blue) and DHE (red). Scale bar: 40 μm. (C) RAW264.7 cells were pre-treated with TQ for 30 min, then with 1 μg ml^−1^ LPS for 3 h. Fluorescence imaging of DAPI (blue) and DHE (red). Scale bar: 40 μm. (B,D) The percentage of cells with positive DHE staining. (E) The percentage of cell number divided by the control group. LPS: lipopolysaccharide; TQ: thymoquinone; DAPI: 4′,6-diamidino−2-phenylindole; DHE: dihydroethidium.

**Figure 7.**
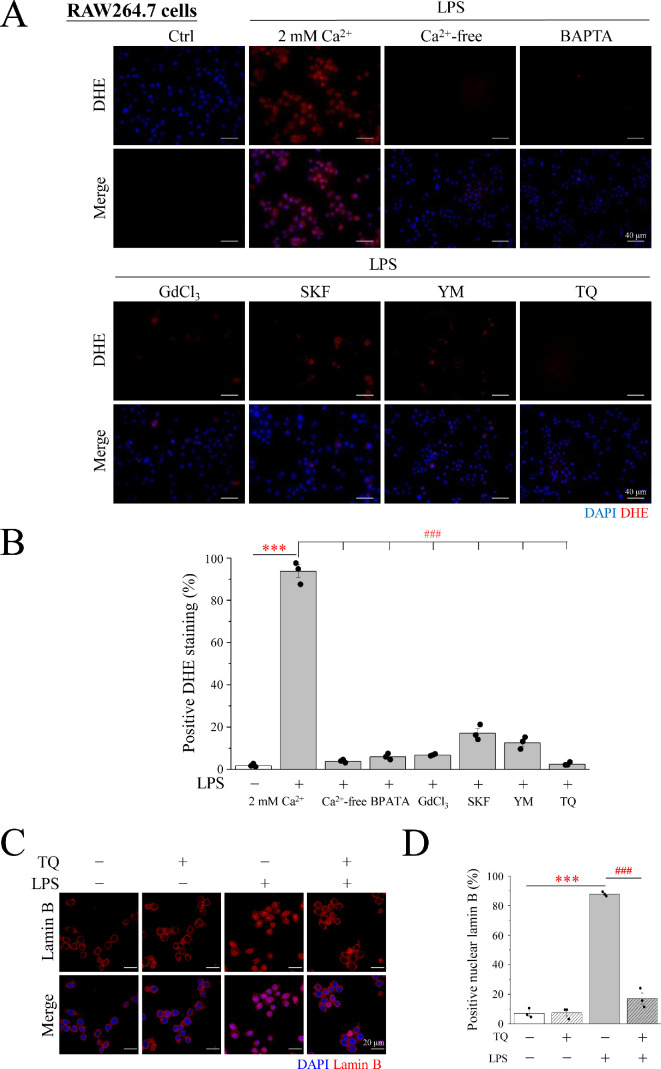
SOCE is essential for cells to perform superoxide anion in METosis in RAW264.7 cells. (A) RAW264.7 cells were treated with 1 μg ml^−1^ LPS for 3 h in Ca^2+^-free (0 mM) or 2 mM Ca^2+^ medium. In addition, pre-treatment with BAPTA-AM, GdCl_3_, SKF96365, YM58483 or TQ was carried out for 30 min, then with LPS for 3 h. Fluorescence imaging of DAPI (blue) and DHE (red). Scale bar: 40 μm. (B) The percentage of cells with positive DHE staining. (C) RAW264.7 cells were pre-treated with TQ for 30 min, then with 1 μg ml^−1^ LPS for 3 h. Fluorescence imaging of DAPI (blue) and lamin B (red). Scale bar: 40 μm. (D) The percentage of RAW264.7 cells with positive nuclear lamin B staining. SOCE: store-operated Ca^2+^ entry; LPS: lipopolysaccharide; DAPI: 4′6-diamidino-2-phenylindole; TQ: thymoquinone; DHE: dihydroethidium.

**Figure 8.**
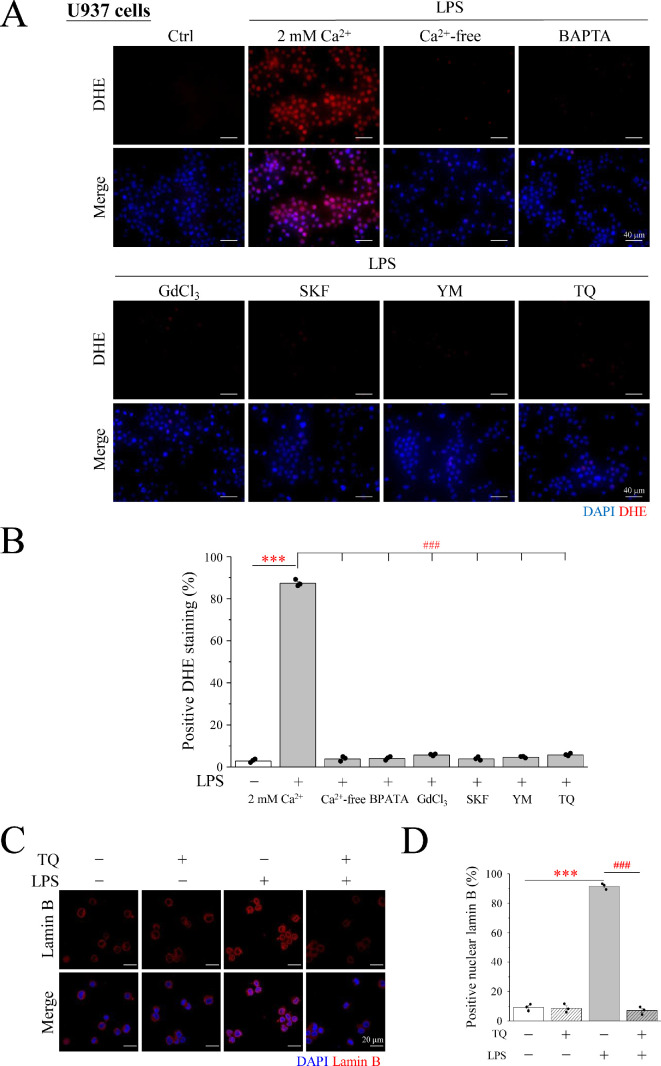
SOCE is essential for cells to produce superoxide anions in METosis in U937 cells. (A) U937 cells were treated with 1 μg ml^−1^ LPS for 3 h in Ca^2+^-free (0 mM) or 2 mM Ca^2+^ medium. In addition, pre-treatment with BAPTA-AM, GdCl_3_, SKF96365, YM58483 or TQ was carried out for 30 min, then with LPS for 3 h. Fluorescence imaging of DAPI (blue) and DHE (red). Scale bar: 40 μm. (B) The percentage of cells with positive DHE staining. (C) U937 cells were pre-treated with TQ for 30 min, then with 1 μg ml^−1^ LPS for 3 h. Fluorescence imaging of DAPI (blue) and lamin B (red). Scale bar: 40 μm. (D) The percentage of U937 cells with positive nuclear lamin B staining. SOCE: store-operated Ca^2+^ entry; LPS: lipopolysaccharide; DAPI: 4′,6-diamidino-2-phenylindole; TQ: thymoquinone; DHE: dihydroethidium.

Macrophages generate a substantial amount of ROS through the action of NOX, which catalyse METosis. DCFDA, the most well-known fluorescent substance used for testing ROS, was used to determine whether ROS production occurred in LPS-induced METs (figure 5). Macrophages showed an obvious green DCFDA fluorescent signal upon LPS induction (figure 5A,B). The relationship between Ca^2+^ and ROS production was verified using the intracellular Ca^2+^ chelator BAPTA-AM. After the addition of BAPTA, the LPS-induced DCFDA signal caused by LPS was significantly diminished (figure 5C,D). This observation establishes that Ca^2+^ is a pivotal mediator driving ROS production. However, ROS comprise a collection of substances and superoxide anions are produced when NOX oxidizes NADPH. Therefore, DHE, a superoxide anion detector, was applied to measure the expression of superoxide anions in METs in RAW246.7 cells. We found that LPS induced a significant increase in the DHE fluorescent signal (figure 6A,B), which shows that superoxide anions can be enhanced by LPS stimulation. TQ, a well-established superoxide anion scavenger, was used to elucidate this phenomenon. The optimal concentration of TQ and cytotoxic effects were examined in RAW246.7 cells. After a 3 h treatment regimen, the addition of 2 μM TQ caused a significant attenuation of the DHE signal compared with the DHE signal intensity seen in the LPS induction group (figure 6C). Notably, when the concentration was increased to 5 μM, a significant decrease was observed, making the signal almost imperceptible (figure 6D). Therefore, 5 μM TQ was determined as the optimal concentration effective in scavenging intracellular superoxide anions while maintaining cell viability (figure 6E). The LPS-induced group exhibited a marked surge in superoxide anion levels, as evidenced by the conspicuous upswing in the DHE signal, in stark contrast to the control group. However, the generation of superoxide anions induced by LPS was not observed in the Ca^2+^-free media. After treatment with BAPTA-AM, GdCl_3_, SKF96365 or YM58483 in a 2 mM Ca^2+^ medium, the red signal of LPS-induced DHE was significantly diminished in RAW246.7 cells (figure 7A,B). This phenomenon was also observed in U937 cells (figure 8A,B). This indicated that Ca^2+^ promotes the generation of superoxide anions in LPS-induced MET through store-operated Ca^2+^ channels. LPS-induced superoxide anion production and METosis were also examined herein. After the cells were treated with TQ, nuclear lamin B staining was significantly inhibited, indicating that the nuclear membrane was intact; that is, METosis did not occur (figure 7C). The results showed that the superoxide anion-scavenging ability of TQ significantly reduced LPS-induced MET production by 85%, providing compelling evidence that superoxide anions are necessary for MET production (figure 7D). These results were also observed in U937 cells, a human monocyte cell line that differentiates into macrophages (figure 8C,D). This illustrates the importance of Ca^2+^ in METosis caused by superoxide anions under LPS stimulation. Finally, the protein expression of NOX1 and NOX2 was examined by Western blotting (electronic supplementary material, figure S2); additionally, PMA and LPS did not affect the expression of NOX1 and NOX2 but mainly affected the activity of NOX through Ca^2+^ signalling.

## 4. Discussion

METs represent a fascinating facet of the immune system, revealing a novel mechanism through which macrophages orchestrate intricate defences against microbial invaders. The dynamic interplay between METs and immune cells and their roles in various pathological conditions have garnered considerable attention in the fields of immunology and infectious diseases. In the present study, we aimed to elucidate the role of Ca^2+^ in LPS-induced METosis. It became evident that employing digitonin instead of Triton X-100 in immunofluorescent staining, coupled with the nuclear membrane indicator lamin B, proved efficacious in distinguishing the occurrence of MET (figure 1). Recognizing PMA as an artificial product, LPS, a pivotal component of the outer cell membrane of Gram-negative bacteria, was used to induce MET herein (figures 1 and 2; electronic supplementary material, figure S1). Furthermore, we investigated whether Ca^2+^ triggered METosis. The LPS-treated group had obvious Ca^2+^ oscillations; the amplitude of LPS was consistent with the higher frequency, and oscillation was observed even in the Ca^2+^-free medium (figure 3F–I). However, PMA induced Ca^2+^ oscillations only in the 2 mM Ca^2+^ medium (figure 3A–D). Additionally, PMA can only induce Ca^2+^ oscillations in 2 mM Ca^2+^ medium instead of Ca^2+^-free medium. However, LPS can simultaneously activate the release of Ca^2+^ from the ER into the cytoplasm and the influx of extracellular Ca^2+^. Therefore, the scale of Ca^2+^ oscillations caused by LPS in the 2 mM Ca^2+^ medium was significantly larger than that in the Ca^2+^-free medium (figure 3). Concomitantly, it was found that the contribution of Ca^2+^ influx to the increase in cytoplasmic Ca^2+^ is significantly greater than that of ER-releasable Ca^2+^.

Notably, BAPTA-AM significantly reduced the LPS-induced ROS production and METosis (figures 4 and 5), demonstrating the importance of Ca^2+^ in ROS-mediated METosis. Furthermore, Ca^2+^ influx in the form of SOCE played a key role in Ca^2+^-induced METosis (figure 4). We also demonstrated that LPS triggers the production of superoxide anions (figure 6). Again, Ca^2+^ influx in the form of SOCE is also the main factor in LPS-induced superoxide anion-mediated METosis in RAW264.7 (figure 7) and U937cells (figure 8). Previous studies divided METosis into NOX-dependent- and NOX-independent pathways. Herein, it was determined that PMA and LPS mainly regulate METosis through a NOX-dependent pathway, whereas METosis caused by Ca^2+^ ionophores (A23187 and ionomycin) mainly regulates METosis through a NOX-independent pathway. Ca^2+^ signalling is the main signalling pathway in cells activated by PMA and LPS. The findings of the present study provide robust evidence that Ca^2+^ signalling, especially SOCE, is the main and indispensable signalling pathway for PMA and LPS through a NOX-dependent pathway to cause METosis.

Considering the challenges associated with conventional electron microscopy for identifying MET, the present study proposes an ideal and feasible immunofluorescence staining approach aimed at distinctly characterizing METosis, while mitigating the limitations inherent in electron microscopy. This innovative method involves the substitution of Triton X-100 with digitonin for immunofluorescence staining of lamin proteins in the nuclear lamina. This improved immunofluorescence staining method not only improves the specificity and accuracy of MET identification but also avoids the use of expensive and complex electron microscopy, bringing promising advancements to METosis-related research.

## Data Availability

Supplementary material is available online [29].
